# Comparison of Valgus Correction Angle between Patients with Developmental Dysplasia Hip and Normal Volunteers Measured by Three-Dimensional Reconstruction and X-Ray

**DOI:** 10.1155/2020/2049306

**Published:** 2020-05-24

**Authors:** Aobo Zhang, Qing Han, Bingpeng Chen, Chenyu Wang, Xue Zhao, Jincheng Wang

**Affiliations:** ^1^Department of Orthopedics, The Second Hospital of Jilin University, 130000, China; ^2^Department of Plastic and Cosmetic Surgery, The First Hospital of Jilin University, 130000, China; ^3^Department of Endocrinology and Metabolism, The First Hospital of Jilin University, 130000, China

## Abstract

**Methods:**

Bilateral VCA of 50 DDH patients and 56 normal volunteers were measured by Mimics software in the 3D method and X-ray in 2D. Two VCA (the upper VCA and the lower VCA) were measured in both two methods. Every VCA was measured by observer A and observer B for twice separately. The statistical analyses of the differences were calculated among the measurements of the VCA.

**Results:**

The mean value of the upper VCA measured in 3D was 4.95° ± 0.76° in DDH group and 5.56° ± 0.62° in the normal group with significant difference (*t* = −6.457, *p* < 0.01). The VCA of DDH group and normal group measured by 3D was larger than 2D, both the upper VCA and the lower VCA. The differences indicated statistically significant. The mean value of lower VCA was 0.60° smaller than the mean value of upper VCA in normal volunteers. The mean value of the lower VCA was 0.58° larger than the mean value of the upper VCA in DDH patients.

**Conclusions:**

Compared to X-ray, 3D reconstruction technology is more accurate without conventional limitations. The lower VCA of DDH patients should be regarded as the femoral intramedullary guide angle in TKA, especially for patients with femoral deformities.

## 1. Introduction

**Figure 1:**
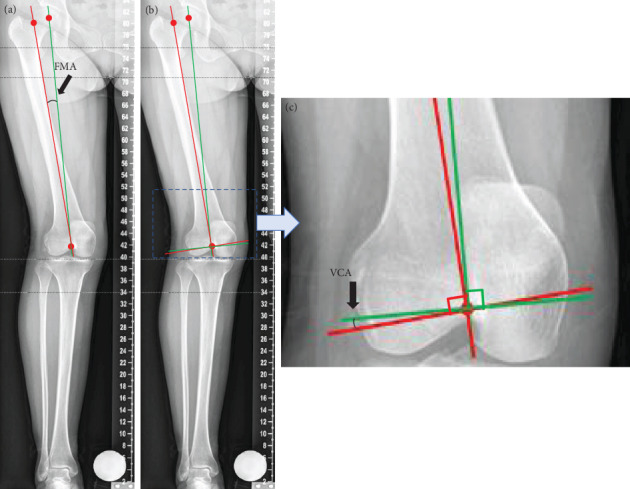
The relation between FMA and VCA. (a) Determining the FMA by the angle between femoral mechanical axis and femoral anatomical axis. (b) Determining the VCA by the lines perpendicular to femoral mechanical axis and femoral anatomical axis. (c) Partial enlarged details show that the FMA is equal to VCA.

Accurate restoration of limb alignment is one of the crucial preconditions to the success of total knee arthroplasty (TKA) [1–4]; otherwise, it can cause loosening, instability, and polyethylene wear during the long period of usage [5]. In previous research, a TKA malalignment of more than 3 degrees can significantly increase the risk of failure [6]. It is essential to achieve a neutral alignment, which needs the femoral and tibial components to be implanted perpendicularly to the femoral and tibial mechanical axis [7]. In contrast, it is more difficult to do distal femoral resection than proximal tibial resection, since the valgus correction angle (VCA) is hard to measure during surgery [8]. In most TKA, the intramedullary guide needs to be aligned with the femoral anatomical axis. The VCA should be equal to the femoral mechanical anatomical angle (FMA), the angle between the femoral mechanical axis and the femoral anatomical axis (Figure 1). In some particular cases, when the femur is in bowing deformity, the distal femoral anatomical axis is considered to be the direction of the intramedullary guide. Whatever the case, the application of a preoperative measurement is necessary which can provide a reference for intraoperative measurement to ensure accurate operation. However, a fixed 5° or 6° VCA is usually applied to uncomplicated TKA surgeries routinely at present; it ignores the individual differences of patients. The results in some studies demonstrate that the VCA of different patients have great variations, and it was affected by multiple factors. A fixed VCA can cause unacceptable planning errors in many cases [1, 9–11]. For this reason, it is important to measure the VCA of every patient before the operation to comprehensively evaluate physical condition and prepare for surgical planning.

Conventional 2D radiograph imaging is usually used for the measurements of VCA. It has the advantages of convenience, low cost, and a low dose of radiation [5]. However, the measurements are sensitive to patients, since the requirement of orientation and position is specific. When the femoral bowing is in an excessive degree, which is very common in DDH patients, the rotational conditions will influence the measurement [2, 4]. Another main shortcoming of conventional radiography is that the marker lines used for VCA calculation are 2D projections of anatomical 3D bone structures; it is considered with biases since the VCA is actually the space angle [4]. In this research, we decided to utilize 3D reconstruction technology to measure VCA and compared with the results measured by radiograph. Beyond that, 2 VCA (the upper VCA and the lower VCA) were measured to suit more clinical situations. The upper VCA was defined as the angle between the femoral mechanical axis and the femoral anatomical axis. The lower VCA was defined as the angle between the femoral mechanical axis and the distal femoral anatomical axis. The differences between the 2 VCA can be the evaluation of the accuracy and sensitivity of these two methods.

## 2. Materials and Methods

### 2.1. Patients and Groups

This study was approved by our institutional internal review board (no. 175 in 2018). All patients and volunteers enrolled in the study provided written informed consent. 51 DDH patients and 58 normal volunteers attended in this study. 1 DDH patient was excluded due to the age. 2 volunteers were excluded due to the history of femoral fracture. At last, 50 DDH patients and 56 volunteers were adopted in this study. The inclusion criteria were listed as follows:

DDH patients:

1. age: >18 years
2. diagnosed as DDH patients without the history of femoral fracture

Normal volunteers:

1. age: >18 years
2. volunteers without the history of femoral fracture
3. volunteers without congenital malformation

Examinations were carried out on a Philips iCT 256 CT scanner at 156 mA and 120 kVp with a slice thickness of 0.602 mm. The CT scanning data and X-ray data were collected from 50 DDH patients and 56 normal volunteers. The DDH group consisted of DDH patients with a mean age of 53 years (age range, 23-84). The normal group consisted of normal volunteers with a mean age of 20 years (age range, 18-21). Mimics software (v19.0, Materialise, Belgium) was used to analyzed and measured VCA on 3D. The VCA on 2D could be directly measured by X-ray photographs. The upper VCA and the lower VCA were measured in both 3D and 2D methods.

### 2.2. Measurement of VCA in 3D

#### 2.2.1. Reconstruction of 3D Femur

**Figure 2:**
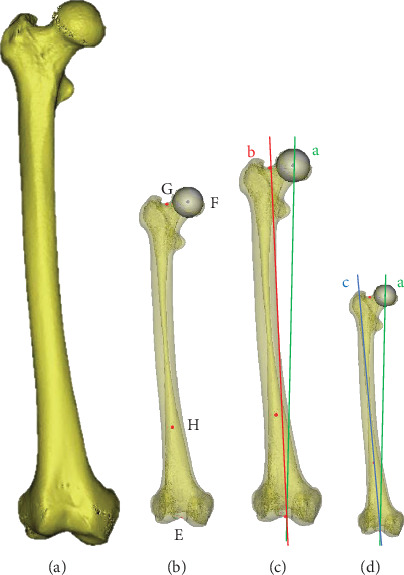
The upper VCA and the lower VCA on the 3D reconstruction model. (a) 3D reconstruction model of femur. (b) Point E: the center of the cortical bone of intercondylar notch in the most distal cross-section. Point F: the center of the femoral head point G: the center point of femoral neck isthmus. Point H: the midpoint of distal one-third femoral length. (c) The angle between the femoral mechanical axis (line a) and femoral anatomical axis (line b) is the upper VCA. (d) The angle between the femoral mechanical axis (line a) and the distal femur anatomical axis (line c) is the lower VCA.

The CT scanning imagines were imported into Mimics software to produce the model in 3D. Firstly, a certain threshold value needs to be set to distinguish the bone from muscles, soft tissues, and ligaments. In this study, the minimum threshold value was selected at 190-210 (average 200) to obtain distinct femur outlines. Then, the femur was marked to calculate the 3D model to complete the reconstruction of the 3D model (Figure 2(a)).

#### 2.2.2. Locating Marker Points and Lines

The upper VCA was equal to FMA, refer to the angle between the femoral mechanical axis and the femoral anatomical axis. The mechanical axis was defined as the line joining the femoral head center and the intercondylar notch. The femoral anatomical axis was defined as the line joining the femoral intercondylar notch and femoral neck isthmus [12]. Firstly, the center of the cortical bone of intercondylar notch in the most distal cross-section was selected as point E. The vertex of the intercondylar notch was the entry point of the intramedullary guide. The femoral head was supposed as a spherical model after 4 different points had been selected on it. The center of the sphere was point F. Point G was defined as the center point of the femoral neck isthmus. Point H was defined as the midpoint of the distal one-third femoral length (Figure 2(b)). The line (a) joining point E and point F was defined as the femoral mechanical axis. The line (b) joining point E and point G was defined as the femoral anatomical axis, which was the line through the femoral diaphysis [13]. The angle between line (a) and line (b) was defined as the upper VCA (Figure 2(c)).

The lower VCA was defined as the angle between the femoral mechanical axis and the anatomical axis of the distal femur. The line (c) joining point E and point H was defined as the anatomical axis of the distal femur. The angle between line (a) and line (c) was defined as the lower VCA (Figure 2(d)).

The angles were measured by the tools in Mimics Research 19.0 and carefully recorded.

### 2.3. Measurement of VCA in 2D

**Figure 3:**
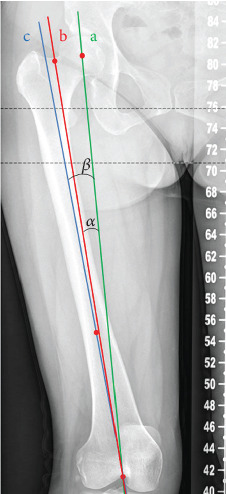
The upper VCA and the lower VCA on X-ray. Line a: femoral mechanical axis. Line b: femoral anatomical axis. Line c: distal femur anatomical axis. ∠*α*: the upper VCA. ∠*β*: the lower VCA.

The marker lines and points were exactly the same as the measurement in 3D. The upper VCA and lower VCA could be directly measured on the radiograph and carefully recorded (Figure 3).

### 2.4. Statistics

Statistical analyses were performed with SPSS software (v21.0, IBM, America). Interobserver and intraobserver reliability were evaluated by intraclass correlation coefficient (ICC), 95% confidence interval (CI) with a 2-way random model, absolute agreement for single measures. The data was presented by mean ± SD, and the *P* values < 0.05 were considered to be significant. The values of VCA measured by the different methods in the DDH group and the normal group were analyzed by paired samples *t*-test. The values of VCA measured by the same method between the DDH group and the normal group were analyzed by independent samples *t*-test.

## 3. Results

### 3.1. Interobserver and Intraobserver Reliability

Normal distribution test and estimated distribution parameters were analyzed; all statistics were in the line with the normal distribution. There were high interobserver and intraobserver reliability for 2 observers (Table 1). The final result of VCA was the average of 2 observers (Table 2).

### 3.2. Comparison of VCA in DDH Group

#### 3.2.1. Comparison of VCA in 3D

The mean value of the upper VCA was 4.95° ± 0.76°; the mean value of the lower VCA was 5.52° ± 1.85°. The paired samples *t*-test was used to compare the differences between the upper VCA and the lower VCA, *t* = −3.690, *p* < 0.001.

#### 3.2.2. Comparison of VCA in X-Ray

The mean value of the upper VCA was 4.03° ± 1.08°; the mean value of the lower VCA was 4.31° ± 2.11°. The paired samples *t*-test was used to compare the differences between the upper VCA and the lower VCA, *t* = −1.388, *p* = 0.168.

#### 3.2.3. Comparison of VCA between 3D and X-Ray

The paired samples *t*-test was used to compare the differences of the upper and the lower VCA between 3D and X-ray, the results were *t* = 13.666, *p* < 0.001 and *t* = 9.916, *p* < 0.001.

### 3.3. Comparison of VCA in Normal Group

#### 3.3.1. Comparison of VCA in 3D

The mean value of the upper VCA was 5.56° ± 0.62°; the mean value of the lower VCA was 4.96° ± 0.88°. The paired samples *t*-test was used to compare the differences between the upper VCA and the lower VCA, *t* = 6.171, *p* < 0.001.

#### 3.3.2. Comparison of VCA in X-Ray

The mean value of the upper VCA was 5.21° ± 0.68°; the mean value of the lower VCA was 2.87° ± 1.20°. The paired samples *t*-test was used to compare the differences between the upper VCA and the lower VCA, *t* = 20.917, *p* < 0.001.

#### 3.3.3. Comparison of VCA between 3D and X-Ray

The paired samples *t*-test was used to compare the differences of the upper and the lower VCA between 3D and X-ray; the results were *t* = 11.597, *p* < 0.001 and *t* = 19.549, *p* < 0.001.

### 3.4. Comparison of VCA between DDH Group and Normal Group

#### 3.4.1. Comparison of VCA in 3D

The independent samples *t*-test was used to compare the differences of the upper and the lower VCA between DDH group and normal group; the results were *t* = −6.457, *p* < 0.001 and *t* = 2.761, *p* = 0.007.

#### 3.4.2. Comparison of VCA in X-Ray

The independent samples *t*-test was used to compare the differences of the upper and the lower VCA between DDH group and normal group, the results were *t* = −9.396, *p* < 0.001 and *t* = 5.987, *p* < 0.001.

## 4. Discussion

Nowadays, most surgeons still use fixed VCA of 5° or 6°in different patients during conventional TKA. In previous studies, Mullaji et al. reported that 56% of 503 knees undergoing TKA had VCA outside the normal range of 5°-7° [14]. Shetty et al. reported that 38% of 100 knees undergoing TKA had VCA outside the range of 5°-7° [15]; Palanisami et al. reported 31% of 227 knees undergoing TKA had VCA outside 5°-7° [16]. The results of our research measured by 3D indicated that 18% of 112 knees of normal volunteers had VCA outside 5°-7°; 56% of 100 knees of DDH patients had VCA outside 5°-7°. The difference between normal volunteers and DDH patients was obviously huge, which indicated that the VCA of DDH patients varied with more abnormal values. Although the gender and lateral differences are not significant, variations in the anatomy of femora still influence VCA such as femoral and tibial bowing and neck-shaft angle [17]. DDH patients usually have anatomical deformities and abnormalities of femora [18], such as a severe deformity of the femoral head and excessive femoral bowing of femoral shaft. It indicates that situations vary hugely and individual measurement in preoperative design is required. Our study firstly focused on the VCA of DDH patients and proposed a new method to measure the VCA. In this research, the VCA of normal volunteers and DDH patients were measured in a new method of 3D to perform accurate and comprehensive treatment for each individual patient.

**Figure 4:**
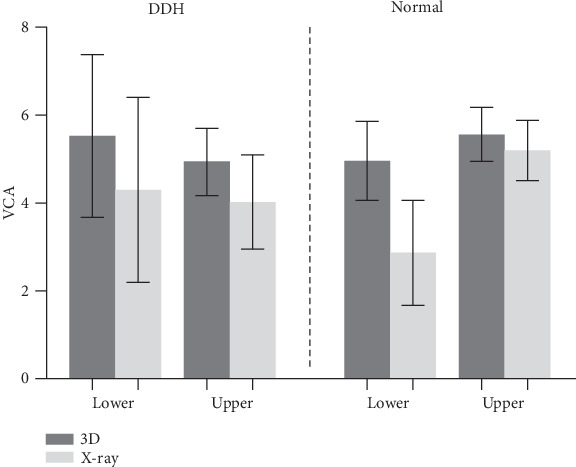
Bar graph of mean (±SD) VCA in DDH and normal group measured by 3D and X-ray. Error bars denote SDs. All these four groups have significant differences (*p* < 0.05).

In clinical routine, the analysis and measurement of VCA rely on radiograph or CT scan [19]. However, the measurement of plane goniometry is projected angles, which may increase errors. Besides, measurements in radiographs are influenced by the positions, especially in DDH patients with deformities of limbs. When an excessive degree of femoral bowing is presented, the radiographic measurements can be affected by rotational degree [4]. For the patients with tibial or femoral torsion, the accurate profile of the tibial plateau is hard to distinguish. Conventional CT scan data can provide these patients with accurate preoperative measurements, but it requires patients to maintain special body orientation during scanning [5, 20, 21]. Compared with the methods in 2D, 3D reconstruction technology based on CT can be more intuitive to analyze and calculate the clinical parameters in any plane and angle as needed. In DDH patients, the marker lines and points are hard to define in 2D, because of the anatomical deformities. In this research, 3D technology was applied to avoid these disadvantages and measure the VCA of normal volunteers and DDH patients accurately. A comparison with radiography was also made. The results indicated that 3D measurements of VCA were significantly larger than 2D in both DDH patients and normal volunteers. The VCA of DDH patients measured by 3D was larger than 2D at a mean of 0.92° (the upper VCA) with significant differences (*t* = 11.597, *p* < 0.001) and 1.22° (the lower VCA) with significant differences (*t* = 19.549, *p* < 0.001). The VCA of normal volunteers measured by 3D was larger than 2D at a mean of 0.35° (the upper VCA) with significant differences (*t* = 13.666, *p* < 0.001) and 2.10° (the lower VCA) with significant differences (*t* = 9.916, *p* < 0.001) (Figure 4). The lack of values in the preoperative measurements may reduce clinical relevance. According to the previous study, one degree of inaccuracy amount to 1-mm bone thickness. In another word, if the angle is 2° outside the range, it will cause the difference between component thicknesses, the gap between bone ends varies by 2 mm [22]. Warakorn et al. reported that the mean value of FMA was 6.46° ± 1.26° in Thailand in 2D method [6]. Kharwadkar et al. reported that the mean value of FMA was 5.4° ± 0.9° in India in 2D method [23]. Mullaji et al. reported that the mean value of FMA was 7.3° ± 1.6° in India in 2D method [14]. Deakin et al. reported that the mean value of FMA was 5.7° ± 1.2° in the USA in 2D method [3]. Bardakos et al. reported that the mean value of FMA was 5.6° ± 1.0° in the UK in 2D method [19]. Chaibi et al. reported that the mean value of the FMA of 20 normal volunteers was 5.3° ± 0.75° in France in 3D method [4]. In this research, the mean value of the FMA of the normal volunteers was 5.6° ± 0.62° in China in 3D method (Table 3). It was proved that the method we used was reproducible since the results were similar with previous studies. Due to the region difference, the value of VCA was still a little different. Chinese people usually have larger obliquity of the knee compare to people in Europe. Previous studies from India and China indicated that the FMA of Asians was larger than Europeans [6]. The results of this research provided reference data for the study of Chinese femoral anatomy. Beyond these, the results of this research indicated that there was no significant difference when X-ray was used to measure the upper and lower VCA of DDH patients (*t* = −1.388, *p* = .168). However, when 3D method was used, there were significant differences between the upper and lower VCA (*t* = −3.690, *p* < 0.001). It proved that the method of 3D was more sensitive, and it could indicate the details that the conventional method could not reveal. For all the above reasons, 3D measurements were more accurate, reliable, and reproducible compared to the measurements in 2D.

**Figure 5:**
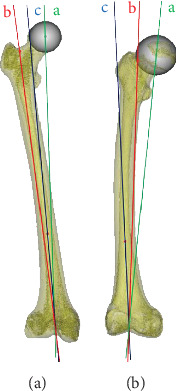
Comparison of the upper VCA and the lower VCA between normal volunteers and DDH patients. (a) The upper VCA is larger than the lower VCA in normal volunteers. (b) The upper VCA is smaller than the lower VCA in DDH patients.

In order to be more accordant with actual situations on clinic, two different VCA (the upper VCA and the lower VCA) were measured in this research. In general, the femora of the patients are in normal shape. The upper VCA is used to confirm and adjust the limb alignment during TKA for most of the patients. However, the femora of some DDH patients are usually abnormal in femoral bowing. In such cases, the intramedullary rod implanted into a distal femur along with the femoral anatomical axis can cause stress concentration and fracture [16]. The lower VCA is more in line with the operation in these cases compared to the upper VCA. The lower VCA is regarded as the femoral intramedullary guide angle, the angle between the intramedullary rod and the femoral mechanical axis. The intersection between these two lines is the entry point of the intramedullary guide. It has been proved that the intramedullary guide has a relationship with femoral bowing [8, 24]. The results in this research indicated that the mean value of lower VCA is 0.60° smaller than the mean value of upper VCA in normal volunteers. On the opposite, the mean value of lower VCA is 0.58° larger than the mean value of the upper VCA in DDH patients (Figure 5). According to the previous study on Korean consecutive osteoarthritis patients, the shape of the proximal femur indicated no relation with the femoral intramedullary guide angle, but the shape of the distal femur obviously had a relation with the femoral intramedullary guide angle [8]. It was thought of as the consequence of abnormal femoral bowing which was very common in osteoarthritis and DDH patients. Therefore, it was important to confirm the deformities of the femur to increase the accuracy of the distal femoral cut in TKA. The results of our research also follow this relation. On the one hand, it proves that the femora of DDH patients usually have anatomical deformities in bowing; on the other hand, we need to discriminate and pay attention to the differences to lay the foundation for the success of the operation.

Our research focused on the different VCA of DDH patients and normal volunteers for the first time. The differences of VCA, both the upper VCA and the lower VCA, between normal volunteers and DDH patients in 3D and 2D were significant. The results of this research proved the method of 3D could maximize accuracy and ensure reproducibility at the same time compared with 2D method. Despite this, the deformities of the femur still affect the VCA and should be paid more attention to further treatment and operation. The individual treatments for patients should be used instead of conventional fixed ways according to different diseases from one to another so that the implant alignment of the lower limbs could be improved during TKA.

## 5. Conclusion

Compared to the radiograph, 3D reconstruction technology is more accurate and reliable without conventional limitations. Compared to the upper VCA, the lower VCA is more suitable for the patients with femoral deformities and should be regarded as the femoral intramedullary guide angle in TKA. We suggest the lower VCA measured by 3D as the reference for preoperative planning, especially for DDH patients.

## Figures and Tables

**Table 1:** Interobserver and intraobserver reliability estimated by intraclass correlation coefficient (ICC).

VCA			ICC (95% CI)	ICC (95% CI)	ICC (95% CI)	ICC (95% CI)
			A1-A2	B1-B2	A1-B1	A2-B2
DDH	3D	Lower	.995 (.992-.966)	.995 (.992-.997)	.992 (.988-.995)	.993 (.990-.995)
		Upper	.976 (.956-.980)	.971 (.957-.980)	.969 (.954-.979)	.968 (.953-.978)
	X-ray	Lower	.986 (.979-.990)	.985 (.978-.990)	.978 (.968-.985)	.978 (.968-.985)
		Upper	.961 (.942-.973)	.958 (.938-.972)	.959 (.940-.972)	.961 (.943-.974)

Normal	3D	Lower	.882 (.833-.917)	.895 (.851-.927)	.892 (.846-.924)	.895 (.851-.926)
		Upper	.761 (.670-.829)	.785 (.701-.847)	.736 (.639-.811)	.725 (.624-.802)
	X-ray	Lower	.974 (.963-.982)	.976 (.966-.984)	.967 (.953-.978)	.967 (.952-.977)
		Upper	.799 (.720-.857)	.795 (.716-.855)	.917 (.882-.942)	.901 (.859-.931)

ICC value: 0 = no correlation, 1 = perfect correlation.

VCA: valgus correction angle; DDH: developmental dysplasia of the hip; ICC: intraclass correlation coefficient.

**Table 2:** The VCA of DDH group and normal group.

Group	Method	VCA	*N*	Range (°)	Mean (°) ± SD (°)
DDH	3D	Upper	100	2.98-7.83	4.95 ± 0.76
DDH	3D	Lower	100	0.83-11.48	5.53 ± 1.85
DDH	X-ray	Upper	100	0.98-6.89	4.03 ± 1.08
DDH	X-ray	Lower	100	-4.34-11.39	4.31 ± 2.11
Normal	3D	Upper	112	3.42-7.00	5.56 ± 0.62
Normal	3D	Lower	112	2.50-7.45	4.97 ± 0.88
Normal	X-ray	Upper	112	2.91-6.87	5.21 ± 0.68
Normal	X-ray	Lower	112	0.36-6.37	2.87 ± 1.20

VCA: valgus correction angle; DDH: developmental dysplasia of the hip.

**Table 3:** Previous measurement of FMA in different regions and methods.

Authors	Region	Method	FMA (mean value ± SD)
Warakorn et al.	Thailand	2D	6.5° ± 1.26°
Kharwadkar et al.	India	2D	5.4° ± 0.9°
Mullaji et al.	India	2D	7.3° ± 1.6°
Deakin et al.	USA	2D	5.7° ± 1.2°
Bardakos et al.	UK	2D	5.6° ± 1.0°
Chaibi et al.	France	3D	5.3° ± 0.75°
This study	China	3D	5.6° ± 0.62°

FMA: femoral mechanical anatomical angle.

## Data Availability

The data supporting the findings of this study are available from the corresponding author upon reasonable request.

## References

[1] Shi X., Li H., Zhou Z. (2017). Individual valgus correction angle improves accuracy of postoperative limb alignment restoration after total knee arthroplasty. *Knee Surgery, Sports Traumatology, Arthroscopy*.

[2] Cherian J. J., Kapadia B. H., Banerjee S., Jauregui J. J., Issa K., Mont M. A. (2014). Mechanical, anatomical, and kinematic axis in TKA: concepts and practical applications. *Current Reviews in Musculoskeletal Medicine*.

[3] Deakin A. H., Basanagoudar P. L., Nunag P., Johnston A. T., Sarungi M. (2012). Natural distribution of the femoral mechanical-anatomical angle in an osteoarthritic population and its relevance to total knee arthroplasty. *The Knee*.

[4] Chaibi Y., Cresson T., Aubert B. (2012). Fast 3D reconstruction of the lower limb using a parametric model and statistical inferences and clinical measurements calculation from biplanar X-rays. *Computer Methods in Biomechanics and Biomedical Engineering*.

[5] Okamoto S., Mizu-uchi H., Okazaki K. (2016). Two-dimensional planning can result in internal rotation of the femoral component in total knee arthroplasty. *Knee Surgery, Sports Traumatology, Arthroscopy*.

[6] Jingjit W., Poomcharoen P., Limmahakhun S., Klunklin K., Leerapun T., Rojanasthien S. (2014). Femoral Mechanical-Anatomical Angle of Osteoarthritic Knees. *Journal of the Medical Association of Thailand*.

[7] Deakin A. H., Sarungi M. (2014). A comparison of variable angle versus fixed angle distal femoral resection in primary total knee arthroplasty. *The Journal of Arthroplasty*.

[8] Kim J. M., Hong S. H., Kim J. M. (2015). Femoral shaft bowing in the coronal plane has more significant effect on the coronal alignment of TKA than proximal or distal variations of femoral shape. *Knee Surgery, Sports Traumatology, Arthroscopy*.

[9] Lee C. Y., Huang T. W., Peng K. T., Lee M. S., Hsu R. W., Shen W. J. (2015). Variability of distal femoral valgus resection angle in patients with end-stage osteoarthritis and genu varum deformity: radiographic study in an ethnic Asian population. *Biomedical Journal*.

[10] Shi X., Li H., Zhou Z., Shen B., Yang J., Pei F. (2016). Comparison of postoperative alignment using fixed vs individual valgus correction angle in primary Total knee Arthroplasty with lateral bowing femur. *The Journal of Arthroplasty*.

[11] Gungor H. R., Ok N., Agladioglu K. (2016). “Outliers” in Osteoarthritic Knees Concerning Distal Femoral Valgus Angle and Femoral Rotation Angle. *The Journal of Arthroplasty*.

[12] Sato T., Koga Y., Sobue T., Omori G., Tanabe Y., Sakamoto M. (2007). Quantitative 3-dimensional analysis of preoperative and postoperative joint lines in total knee arthroplasty: a new concept for evaluation of component alignment. *The Journal of Arthroplasty*.

[13] Nam D., Maher P. A., Robles A., McLawhorn A. S., Mayman D. J. (2013). Variability in the relationship between the distal femoral mechanical and anatomical axes in patients undergoing primary total knee arthroplasty. *The Journal of Arthroplasty*.

[14] Mullaji A. B., Shetty G. M., Kanna R., Vadapalli R. C. (2013). The influence of preoperative deformity on valgus correction angle: an analysis of 503 total knee arthroplasties. *The Journal of Arthroplasty*.

[15] Shetty G. M., Mullaji A., Khalifa A. A., Ray A. (2017). Windswept deformities - an indication to individualise valgus correction angle during total knee arthroplasty. *Journal of Orthopaedics*.

[16] Palanisami D., Iyyampillai G., Shanmugam S., Natesan R., S R. (2016). Individualised distal femoral cut improves femoral component placement and limb alignment during total knee replacement in knees with moderate and severe varus deformity. *International Orthopaedics*.

[17] Mullaji A. B., Marawar S. V., Mittal V. (2009). A Comparison of Coronal Plane Axial Femoral Relationships in Asian Patients With Varus Osteoarthritic Knees and Healthy Knees. *The Journal of Arthroplasty*.

[18] Kotlarsky P., Haber R., Bialik V., Eidelman M. (2015). Developmental dysplasia of the hip: what has changed in the last 20 years?. *World Journal of Orthopedics*.

[19] Bardakos N., Cil A., Thompson B., Stocks G. (2007). Mechanical axis cannot be restored in total knee arthroplasty with a fixed valgus resection Angle. *The Journal of Arthroplasty*.

[20] Yin Y., Zhang L., Hou Z. (2016). Measuring femoral neck torsion angle using femoral neck oblique axial computed tomography reconstruction. *International Orthopaedics*.

[21] Wu P. H., Zhang Z. Q., Fang S. Y. (2016). Preoperative measurement of tibial resection in total knee arthroplasty improves accuracy of postoperative limb alignment restoration. *Chinese Medical Journal*.

[22] Kinzel V., Scaddan M., Bradley B., Shakespeare D. (2004). Varus/valgus alignment of the femur in total knee arthroplasty. Can accuracy be improved by pre-operative CT scanning?. *The Knee*.

[23] Kharwadkar N., Kent R. E., Sharara K. H., Naique S. (2006). 5°to 6° of distal femoral cut for uncomplicated primary total knee arthroplasty : is it safe?. *The Knee*.

[24] Lasam M. P. G., Lee K. J., Chang C. B., Kang Y. G., Kim T. K. (2013). Femoral lateral bowing and varus condylar orientation are prevalent and affect axial alignment of TKA in Koreans. *Clinical Orthopaedics and Related Research*.

